# Diseases of the Stomach

**Published:** 1904-10-22

**Authors:** 


### DISEASES OF THE STOMACH.

**Diagnostic Methods.**—It is often of importance to gain accurate knowledge of the size and position of the stomach. Sievert[1] has observed the effects of small and large amounts of $CO_2$ on the boundaries of the stomach as determined by percussion. With small quantities of $CO_2$, such as are evolved by 15 grains of tartaric acid and 30 grains of bicarbonate of sodium, the true boundaries of the organ can be made out, and this test may be performed while the patient is fasting, as no escape of gas through the pylorus occurs. Large amounts of gas artificially distend and dislocate the organ, mostly downwards and towards the right. This is only of value in cases of gastroptosis. Behrend,[2] however, has shown that this procedure is not without danger, as he has observed three cases, one of gastric ulcer near the cardiac end, and two of carcinoma of the œsophagus, in which death occurred shortly afterwards. The amounts given were 60 grains of tartaric acid and 60 grains of sodium bicarbonate, each in half a tumblerful of water. Lincoln[3] finds an accurate and easy method of determining the size and shape of the stomach is by transillumination through a solution of fluorescein—a naphthalin product consisting of phthalic anhydride, five parts, and resorcin seven parts, heated to 200° C. His method is to previously prepare the patient by giving 10 or more grains of quinine during the day, which heightens the effect. He then, in the evening, introduces a small electric lamp, specially constructed for the purpose, and then gives the patient 30 or 40 grains of sodium bicarbonate in a glass of water to neutralise the stomach contents. Another glass of water is then given, in which are dissolved fluorescein $\frac{1}{8}$ gr., glycerin 1 drachm, sodium bicarb. 20 gr. The outline of the stomach can then be clearly seen in the dark, and any abnormal thickening of the anterior wall can be distinguished. The thickness of the abdominal wall is of no importance. Hartmann[4] finds Weber's test for blood in the fæces valuable in the diagnosis of gastric ulcer and carcinoma. Food, free from meat, must be given for three days previously, and care must be taken not to soil the vessels used with sweat from the operator's fingers, as this gives a fallacious reaction. He finds by this means gastric ulcer may be diagnosed from gastric neurosis, and a positive result, combined with deficiency of HCl, and retention of motor power is almost pathognomonic of carcinoma. Carles[5] finds that the examination of the urine for indican is useful. This substance is derived from indol, which is formed in the small intestine by bacterial action. If HCl is present in large amounts the bacterial activity is diminished, so that indican does not appear in cases of hyperchlorhydria, but is almost always present in the urine when HCl is deficient in cases of gastric fermentation or absent as in carcinoma or fever. Sigel[6] does not find the estimation of fatty acids in the urine of any value. He also has tested and found wanting (1) the "tryptophan reaction," a violet colour which is said to appear when chlorine or bromine are added to the vomit in cases of gastric carcinoma. (2) Salomon's test, which consists in the estimation of the albumen and nitrogen in the stomach washings, a large amount being said to show the presence of carcinoma He thinks, however, Gluzinski's method is valuable in determining the supervention of malignancy in an old ulcer. The method is to wash out the empty stomach and then give two test meals and determine the HCl in the washings. The constant presence of HCl is strong evidence against malignancy. The absence of liver dulness as a sign of perforation has long been recognised as unreliable, but Thornton[7] states that it never disappears from the mid and posterior axillary lines in tympanites. English,[8] however, found in four cases of perforation the liver dulness was not apparent as far back as the mid-axillary line; but in two cases where no perforation was present the resonance was equally extensive. Pearson[9] also has recorded two cases where, without perforation, no dulness was detected in the extreme axillary region. He finds no satisfactory explanation for the phenomenon, but considers that it is common in young women, especially at the catamenia; though often associated with an empty stomach he has seen it when a full diet had been taken. Liver dulness was absent in 33 per cent. of 140 cases of unperforated gastric ulcer.

[1] Edin. Med. Jour., August, 1904, p. 170. [2] Med. Chron., April. 1904, p. 54. [3] Med. Record, April 23, 1904, p. 654. [4] Edin. Med. Jour., August, 1904, p. 169. [5] Practitioner, May, 1904, p. 753 [6] Med. Rec, April 23, 1904, p. 670. [7] Practitioner, May, 1904, p. 753. [8] Ibid. [9] Med. Chron., March 1904, p. 363.

*(To be continued.)*

